# Epidemiology and Outcomes of SARS-CoV-2 Infection or Multisystem Inflammatory Syndrome in Children vs Influenza Among Critically Ill Children

**DOI:** 10.1001/jamanetworkopen.2022.17217

**Published:** 2022-06-15

**Authors:** Steven L. Shein, Christopher L. Carroll, Kenneth E. Remy, Colin M. Rogerson, Casey K. McCluskey, Anna Lin, Alexandre T. Rotta

**Affiliations:** 1 Division of Pediatric Critical Care Medicine, Rainbow Babies, Children’s Hospital, Cleveland, Ohio; 2 Connecticut Children’s Medical Center, Hartford; 3 Department of Pediatrics, Indiana University School of Medicine, Indianapolis; 4 Department of Pediatrics, West Virginia University School of Medicine, Morgantown; 5 Division of Pediatric Hospital Medicine, Stanford University, Palo Alto, California; 6 Division of Pediatric Critical Care Medicine, Duke University, Durham, North Carolina

## Abstract

This cohort study compares the epidemiology and outcomes of patients in the pediatric intensive care unit with SARS-CoV-2–related disease during the first 15 months of the COVID-19 pandemic vs children with critical influenza prior to the pandemic.

## Introduction

When assessing risks of SARS-CoV-2 and the need for public health measures for children, some cite its similarities to influenza.^1,2,3^ However, it is unclear whether pediatric critical illness differs between SARS-CoV-2 and influenza. Therefore, we used the Virtual Pediatric Systems database (VPS)^4^ to compare epidemiology and outcomes of patients in the pediatric intensive care unit (PICU) with SARS-CoV-2–related disease during the first 15 months of the COVID-19 pandemic vs children with critical influenza prior to the pandemic.

## Methods

With institutional review board approval from the Connecticut Children’s Medical Center and a waiver of informed consent owing to this cohort study’s use of already collected data, we queried VPS for PICU patients younger than 18 years with a primary diagnosis of influenza (admitted April 2018 to March 2020) or SARS-CoV-2–related disease (COVID-19 or multisystem inflammatory syndrome in children [MISC]; admitted April 2020 to June 2021). Only US PICUs reporting data in all 13 quarters were included (66 centers). Mean admissions per center per quarter were calculated. Data were compared using χ^2^, Wilcoxon rank-sum, or signed-rank tests; 2-tailed *P* < .05 defined statistical significance. This study followed the STROBE reporting guideline. More details appear in the eAppendix in the Supplement.

## Results

We identified 1561 PICU patients with influenza (aged <6 years: 64%; without comorbidity: 45%) and 1959 with SARS-CoV-2 disease (aged <6 years: 29%; without comorbidity: 55%). More children were admitted per center per quarter with SARS-CoV-2 disease than with influenza (median [IQR], 2.8 [1.4-9.6] vs 2.4 [0.9-4.0]; *P* < .001). Children with SARS-CoV-2 disease had higher risk of mortality, but actual mortality did not differ (Table).

**Table.** Mean Patient Characteristics per Quarter

Characteristic	Patients, mean, No. (%)		*P* value^a^
	With influenza (n = 1561 in 8 quarters)	With SARS-CoV-2–related disease (n = 1959 in 5 quarters)	
Age			
<29 d	2.8 (1.4)	6.0 (1.5)	<.001
29 d to 2 y	66.1 (33.9)	48.6 (12.4)	
2 to 6 y	56.8 (29.1)	60.0 (15.3)	
6 to 12 y	43.5 (22.3)	108.2 (27.6)	
12 to <18 y	26.0 (13.3)	169.0 (43.1)	
Weight, median (IQR), kg	14.8 (10.0-26.4)	38.8 (18.9-70.0)	<.001
Sex			
Female	85.0 (43.6)	162.4 (41.5)	.22
Male	110.1 (56.4)	229.4 (58.6)	
Comorbidities			
Neurologic	63.9 (32.7)	74.8 (19.1)	<.001
Respiratory	34.5 (17.7)	22.8 (5.8)	<.001
Genetic	27.8 (14.2)	28.0 (7.1)	<.001
Cardiac	22.4 (11.5)	33.0 (8.4)	.003
Obesity	4.5 (2.3)	59.4 (15.2)	<.001
Premature	15.9 (8.1)	12.0 (3.1)	<.001
Tracheostomy	9.3 (4.7)	12.8 (3.3)	.03
Oncologic	3.5 (1.8)	14.2 (3.6)	.002
Other	2.3 (1.2)	8.4 (2.1)	.03
Transplantation	2.8 (1.4)	6.6 (1.7)	.61
None identified	87.3 (44.7)	214.0 (54.6)	<.001
PIM-2 score, median (IQR), %	1.0 (0.8-3.1)	1.1 (0.9-1.8)	<.001
Received endotracheal intubation	36.9 (18.9)	49.0 (12.5)	<.001
Received ECMO	3.0 (1.5)	3.8 (1.0)	.17
Died before PICU discharge	4.5 (2.3)	5.4 (1.4)	.05

Abbreviations: ECMO, extracorporeal membrane oxygenation; PICU, pediatric intensive care unit; PIM-2, Pediatric Index of Mortality.

^a^ Statistical analyses done with χ^2^ or Wilcoxon-rank sum tests. For the categorical variables, patient numbers are shown as the mean number and percentage of children per quarter (ie, total number divided by 8 for influenza or by 5 for SARS-CoV-2).

Per quarter, there were twice as many mean admissions from SARS-CoV-2–related disease as influenza, and one-third more children received endotracheal intubation (Figure, A). PICU and hospital length of stay were significantly longer with SARS-CoV-2–related disease (Figure, B). Similar patterns were observed in children with and without identified comorbidities.

**Figure.**
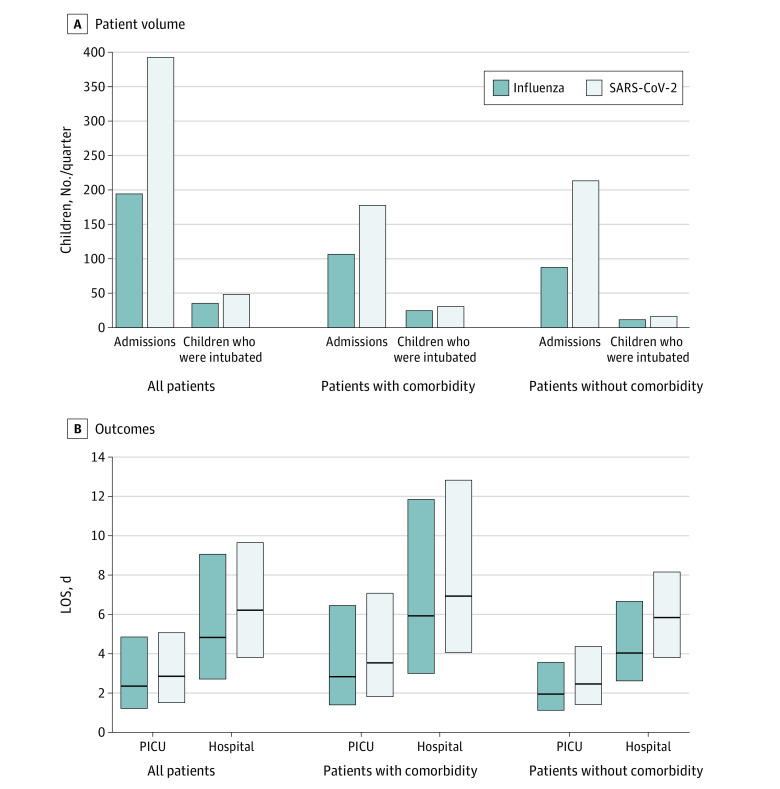
Patient Volumes and Outcomes From Influenza and SARS-CoV-2–Related Disease in 66 Pediatric Intensive Care Units (PICUs) A, The mean numbers of children per quarter admitted to PICUs and undergoing intubation by virus and combordities are shown. B, Lengths of stay (LOS) in PICU and hospital by virus and comorbidities are shown. Bars indicate 25th to 75th percentile, with median indicated by the interior horizontal line.

## Discussion

Among 66 PICUs in the United States, the number of children admitted each quarter with a primary diagnosis of COVID-19 or MISC during the first 15 months of the pandemic was twice as high as that for influenza during the 2 years before the pandemic. Influenza outcomes were observed during a time with no unusual public health measures in place (2018 to early 2020), while those of SARS-CoV-2 occurred while masking, social distancing, and remote schooling occurred. Those measures were sufficient to markedly decrease critical illness from many respiratory viruses, including nearly eliminating influenza admissions to these PICUs.^4^ Without these measures in place for this largely unvaccinated population, SARS-CoV-2 would likely have led to a number of critically ill children several-fold higher than seen with prepandemic influenza as well as more deaths.

Our findings are supported by studies showing increased admissions,^1^ mortality rate,^3^ and absolute numbers of deaths^2^ among children with SARS-CoV-2 vs influenza. However, limitations include our results reflecting the original and Alpha variants and the 2018 to 2020 influenza seasons; applicability to strains of different severities (eg, Delta, Omicron, H1N1) may be limited. Pediatric vaccination and natural immunity were uncommon during the study period, so our findings can best be applied to unimmunized children. We assessed admissions per quarter because influenza seasons and COVID-19 waves have variable onsets and durations, so our data compare overall burden rather than peak seasonal activity. Public health measures^5^ and PICU admissions^6^ have been associated with negative schooling and mental health outcomes that warrant consideration but were unmeasurable in our study. Referring hospitals may have been more likely to transfer patients with SARS-CoV-2 because of their own limited capacity.

In summary, even with pandemic-era public health measures in use, we observed more PICU admissions from SARS-CoV-2 between April 2020 and June 2021 than from influenza during the preceding 2 years. Absence of public health measures when SARS-CoV-2 variants similar to the original and Alpha strains are in circulation would likely lead to a volume of critical illness and death in unimmunized children that is markedly higher than what is typically seen with influenza.

## Notes

Supplement.
eMethods.

## References

[1] Encinosa W, Figueroa J, Elias Y. Severity of hospitalizations from SARS-CoV-2 vs influenza and respiratory syncytial virus infection in children aged 5 to 11 years in 11 US states. JAMA Pediatr. 20222022;176(5):520-522. doi:10.1001/jamapediatrics.2021.6566 35188536 PMC8861895

[2] Laris-González A, Avilés-Robles M, Domínguez-Barrera C, . Influenza vs COVID-19: comparison of clinical characteristics and outcomes in pediatric patients in Mexico City. Front Pediatr. 2021;9:676611. doi:10.3389/fped.2021.676611 34249813 PMC8264261

[3] Piroth L, Cottenet J, Mariet AS, . Comparison of the characteristics, morbidity, and mortality of COVID-19 and seasonal influenza: a nationwide, population-based retrospective cohort study. Lancet Respir Med. 2021;9(3):251-259. doi:10.1016/S2213-2600(20)30527-0 33341155 PMC7832247

[4] Zee-Cheng JE, McCluskey CK, Klein MJ, . Changes in pediatric ICU utilization and clinical trends during the coronavirus pandemic. Chest. 2021;160(2):529-537. doi:10.1016/j.chest.2021.03.004 33727033 PMC7954775

[5] Hawrilenko M, Kroshus E, Tandon P, Christakis D. The association between school closures and child mental health during COVID-19. JAMA Netw Open. 2021;4(9):e2124092. doi:10.1001/jamanetworkopen.2021.24092 34477850 PMC8417763

[6] Odom SL, Sam AM, Tomaszewski B, Cox AW. Quality of educational programs for elementary school-age students with autism. Am J Intellect Dev Disabil. 2022;127(1):29-41. doi:10.1352/1944-7558-127.1.29 34979035

