# Supplementary material for: Epidemiology and Outcomes of SARS-CoV-2 Infection or Multisystem Inflammatory Syndrome in Children vs Influenza Among Critically Ill Children
Source: JAMA Netw Open. 2022 Jun 15;5(6):e2217217. doi: 10.1001/jamanetworkopen.2022.17217 (PMC9201678; doi:10.1001/jamanetworkopen.2022.17217)
Supplement: Supplement. — eMethods. [file jamanetwopen-e2217217-s001.pdf]

Supplemental Online Content

Shein SL, Carroll CL, Remy KE, et al. Epidemiology and outcomes of SARS-CoV-2 infection or multisystem inflammatory syndrome in children vs influenza among critically ill children. *JAMA Netw Open.* 2022;5(6):e2217217. doi:10.1001/jamanetworkopen.2022.17217

eMethods.

This supplemental material has been provided by the authors to give readers additional information about their work.

© 2022 Shein SL et al. *JAMA Network Open.*

eMethods.

With approval from the Institutional Review Board of Connecticut Children's Medical Center including waiver of consent, we queried the VPS database for PICU patients <18 years old with a primary diagnosis of influenza or SARS-CoV-2-related disease (acute COVID-19 or MISC). VPS collects standardized, quality-controlled data to enable benchmarking between participating PICUs and support research. VPS neither endorsed nor restricted our interpretation of these data. Data were collected for children admitted between April 2018 and June 2021. VPS provides admission date by quarter, and only United States PICUs reporting data in all 13 quarters were included (n=66 centers). Because influenza was extremely rare after March 2020 (n=7 children between April 2020 and June 2021) and SARS-CoV-2 disease was extremely rare before April 2020 (n=4), the two analyzed study groups were children admitted from April 2018 to March 2020 with influenza and children admitted from April 2020 to June 2021 with SARS-CoV-2.

We collected demographics, Pediatric Index of Mortality (PIM-2) scores, comorbidities, procedures, and outcomes. Co-morbidities were determined using secondary diagnoses included in the VPS dataset for each subject. For each center, we calculated the average number of children admitted with influenza or with SARS-CoV-2 disease per quarter. Data were compared using chi squared, Wilcoxon rank-sum, or signed-rank tests using SigmaPlot v12.5 (Systat; San Jose, CA). A p-value < 0.05 defined statistical significance. This manuscript is reported in line with the STROBE reporting guidelines.

© 2022 Shein SL et al. *JAMA Network Open.*
